# Structural Insights Into DNA Repair by RNase T—An Exonuclease Processing 3′ End of Structured DNA in Repair Pathways

**DOI:** 10.1371/journal.pbio.1001803

**Published:** 2014-03-04

**Authors:** Yu-Yuan Hsiao, Woei-Horng Fang, Chia-Chia Lee, Yi-Ping Chen, Hanna S. Yuan

**Affiliations:** 1 Institute of Molecular Biology, Academia Sinica, Taipei, Taiwan, Republic of China; 2 Department of Biological Science and Technology, National Chiao Tung University, Hsinchu, Taiwan, Republic of China; 3 Department of Clinical Laboratory Sciences and Medical Biotechnology, College of Medicine, National Taiwan University, Taipei, Taiwan, Republic of China; 4 Graduate Institute of Biochemistry and Molecular Biology, National Taiwan University, Taipei, Taiwan, Republic of China; Brandeis University, United States of America

## Abstract

DNA repair mechanisms are essential for preservation of genome integrity. However, it is not clear how DNA are selected and processed at broken ends by exonucleases during repair pathways. Here we show that the DnaQ-like exonuclease RNase T is critical for *Escherichia coli* resistance to various DNA-damaging agents and UV radiation. RNase T specifically trims the 3′ end of structured DNA, including bulge, bubble, and Y-structured DNA, and it can work with Endonuclease V to restore the deaminated base in an inosine-containing heteroduplex DNA. Crystal structure analyses further reveal how RNase T recognizes the bulge DNA by inserting a phenylalanine into the bulge, and as a result the 3′ end of blunt-end bulge DNA can be digested by RNase T. In contrast, the homodimeric RNase T interacts with the Y-structured DNA by a different binding mode via a single protomer so that the 3′ overhang of the Y-structured DNA can be trimmed closely to the duplex region. Our data suggest that RNase T likely processes bulge and bubble DNA in the Endonuclease V–dependent DNA repair, whereas it processes Y-structured DNA in UV-induced and various other DNA repair pathways. This study thus provides mechanistic insights for RNase T and thousands of DnaQ-like exonucleases in DNA 3′-end processing.

## Abstract 2

Structure analysis of the exonuclease RNase T reveals that it also functions in DNA repair pathways where it binds and processes bulge, bubble, and Y-structured DNA to trim the DNA 3′ ends.

## Author Summary

DNA repair relies on various enzymes, including exonucleases that bind and trim DNA at broken ends. However, we know little about how an exonuclease precisely selects and trims a DNA broken end in specific repair pathways. In this study, the enzyme RNase T, previously known for its involvement in processing RNA substrates, is shown to also possess DNase activity. RNase T is a DnaQ-like exonuclease and is characterized in this work as the exoDNase responsible for trimming the 3′ ends of structured DNA in various DNA repair pathways. Based on the high-resolution crystal structures of RNase T-DNA complexes, an insightful working model is provided showing how RNase T processes bulge, bubble, and Y- structured DNA in various DNA repair pathways. RNase T thus represents a unique structure-specific exonuclease with multiple functions not only in processing 3′ overhangs of duplex RNA during RNA maturation, but also in processing 3′ ends of bubble, bulge, and Y-structured DNA during DNA repair. These findings advance our understanding of the precise function of an exonuclease in DNA repair and suggest possible roles for thousands of members of DnaQ superfamily exonucleases in DNA repair and replication.

## Introduction

It is well known that DNA repair mechanisms maintain genomic integrity and are essential for cell survival. Damaged DNA can be restored by a variety of DNA repair processes, such as direct reversal, base excision, nucleotide excision, mismatch, and recombination repair pathways [1]. Although diverse proteins play different roles in these pathways, DNA repair is generally accomplished by a coordinated effort via several types of DNA enzymes, including endonucleases that nick DNA near the damaged site, exonucleases that trim DNA from the broken end, helicases that unwind duplex DNA, polymerases that make new strand DNA with correct sequences, and ligases that seal the restored DNA strands. Among all these DNA enzymes, the molecular functions of exonucleases, which bind at the 3′ or 5′ end of DNA and cleave one nucleotide at a time, are least understood. How they select, rather than randomly bind to, a broken end of DNA and process it up to the site for the next-step processing remains to be investigated.

Here we use the bacterial exonuclease RNase T as a model system to study the processing of DNA in various DNA repair pathways. RNase T is a member of the DnaQ-like 3′–5′ exonucleases with a DEDDh domain that contains four acidic DEDD residues (D23, E25, D125, and D186) for binding of two magnesium ions, and one histidine residue (H181) for functioning as the general base in the active site for the hydrolysis of the 3′-terminal phosphodiester bond of a nucleic acid chain [2]. The family of DnaQ-like exonucleases constitutes thousands of members, all with exonuclease activity either processing RNA during RNA maturation, interference, and turnover, or processing DNA during DNA replication, degradation, repair, and recombination. A number of the DnaQ-like exonucleases have been shown to play a role in DNA repair. Usually the DEDDh domain can be linked to a DNA polymerase domain for proofreading during DNA replication, such as the DnaQ domain of the ε subunit of *E. coli* pol III holoenzyme and the exonuclease domain of human pol δ, ε, and γ [3]. Mutations in or deletion of the proofreading 3′ exonuclease domain for these polymerases are either lethal or induce high mutation rates and high incidence of cancers [4]. The DEDDh domain can also be linked to a helicase domain and functions in processing of broken DNA strands during DNA repair and recombination, such as that of human WRN [5]. Mutations in the DEDDh exonuclease domain of WRN are associated with Werner syndrome that results in premature aging and increased risk of cancer [6].

However, most of the DEDDh domain functions as an autonomous protein and is not linked to a polymerase or a helicase domain. Some of these exonucleases participate in DNA 3′-end processing in DNA repair, such as ExoI and ExoX from *E. coli* and TREX1 and TREX2 from human [7]–[9]. ExoI and ExoX are monomeric enzymes that digest single-stranded DNA in mismatch and DNA recombination repair pathways [10]–[12], whereas the human TREX1 and TREX2 are dimeric enzymes, likely processing single-stranded DNA in mammalian cells [13],[14]. Mutations in TREX1 are linked to the autoimmune diseases Aicardi-Goutieres syndrome and systemic lupus erythematosus, probably due to the accumulation of nonprocessed intermediate DNA during replication and repair pathways [15]–[17]. The crystal structures of ExoI [18], TREX1 [19], and TREX2 [20] reveal that they all bear a classical α/β fold of the DEDDh domain; nevertheless, their precise functions in DNA processing remain uncertain.

RNase T has also been implicated in the UV-repair pathways based on the observations that the cells lacking RNase T are less resistant to UV radiation and overexpression of RNase T can rescue the UV sensitivity of the ExoI knockout *E. coli* strain [21],[22]. Yet RNase T was originally recognized as an RNase based on its indispensable role in tRNA 3′-end processing during tRNA maturation [23]. RNase T also performs the final trimming for various stable RNA, including 5S and 23S rRNA [24],[25]. RNase T can digest both DNA and RNA and it has a unique specificity that its exonuclease activity is reduced by a single 3′-terminal C or completely abolished by a dinucleotide 3′-terminal CC in digesting either DNA or RNA, referred to as the C effect [26]. Moreover, its exonuclease activity is inhibited by duplex structures, referred to as the double-strand effect; therefore, a 3′ overhang of a duplex DNA or RNA is only digested near the duplex region by RNase T [26],[27]. Previous crystal structures of RNase T in complex with various single-stranded DNA (3′-terminal G versus C) and double-stranded DNA (1 versus 2 nucleotide 3′ overhang) reveal the structural basis for C effect and double-strand effect [27],[28]. The binding of an uncleavable substrate, such as a single-stranded DNA with a 3′-terminal C or a duplex DNA with a short 2-nucleotide 3′ overhang, induces an inactive conformational change in the active site and thus inactivates the exonuclease activity. Therefore, in digesting a duplex DNA with a 3′ overhang, RNase T can accurately differentiate its cleavable or noncleavable substrates based on the C effect and double-strand effect, and it produces a precise final product of a duplex with a 1-nucleotide (if the last base pair in duplex region is AT) or 2-nucleotide (if the last base pair in duplex region is GC) 3′ overhang if the CC dinucleotide is not present within the 3′ tail, or else it stops at the 3′-CC end. RNase T hence is capable to trim various precursor RNA to produce mature RNA with a precise 3′ overhang depending on the structure and sequence of these precursors: 1 nt for 5S rRNA, 2 nt for 23S rRNA, 4 nt for 4.5S RNA, and 4 nt for tRNA [27],[28].

In fact, RNase T is a more efficient DNase than RNase in that it digests DNA with a 10-fold efficiency as compared to RNA (see figure S2 in [28]), supporting its possible cellular role in DNA processing. However, it is not certain if RNase T indeed processes DNA in DNA repair, and if it does, how it selects and processes its DNA substrates. To determine the molecular function of RNase T, we show here by biochemical and structural approaches that it is a structure-specific DNase capable of digesting intermediate structured DNA during DNA repair. We found that RNase T not only digests bubble and bulge DNA in Endonuclease V (Endo V)–dependent DNA repair but also digests Y-structured DNA in UV-induced DNA repair pathways. The crystal structures of RNase T in complex with a bulge and a Y-structured DNA further demonstrate how this dimeric enzyme elegantly binds and processes these structured DNA molecules in different ways. Our results reveal, for the first time, the precise molecular role of an exonuclease in the 3′ end DNA processing and may hint at the molecular function for other members of DnaQ-like exonucleases.

## Results

### RNase T-Deficient Cells Are Sensitive to DNA Damaging Agents

**Figure 1:**
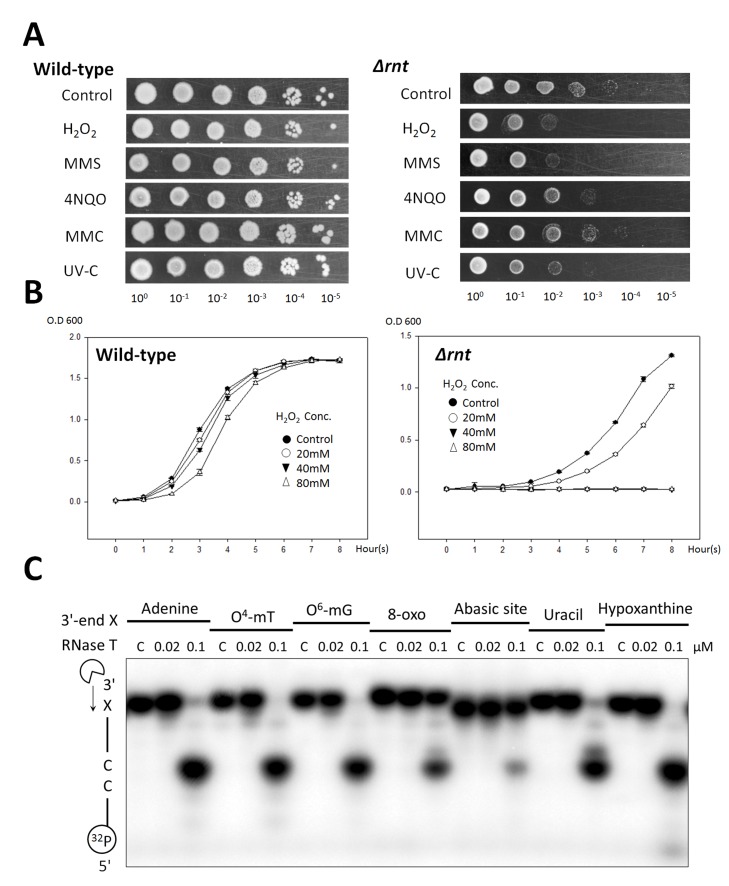
The RNase T knock-out *E. coli* K-12 strains are sensitive to various DNA damaging agents. (A) Wild-type K-12 strains were resistant to chronic doses of various DNA-damaging agents, including hydrogen peroxide (H_2_O_2_), MMS, 4NQO, MMC, and UV-C light. The RNase T-knockout (*Δrnt*) strain had a slow growth phenotype and were sensitive to H_2_O_2_, MMS, 4NQO, and UV-C radiation. (B) Growth curves of wild-type and RNase T knockout cells after acute exposure to H_2_O_2_ (20 to 80 mM) for 20 min. (C) The single-stranded DNA containing a methylated, deaminated, oxidized base or an abasic site at the 3′-terminal end (5′-GAGTCCTATA**X**-3′) were incubated with RNase T in the DNA digestion experiment. RNase T digested single-stranded DNA with a 3′-terminal methylated and deaminated base, including O^4^-methylthymine (O^4^-mT), O^6^-methylguanine (O^6^-mG), uracil, and hypoxanthine (Inosine). However, the single-stranded DNA with a 3′-terminal oxidized base, 8-oxoguanine (8-oxo), or an abasic site was more resistant to RNase T. The cleavage stopped at dincucleotide -CC- because the exonuclease activity of RNase T was inhibited by 3′-terminal CC sequence.

To confirm the possible roles of RNase T in DNA repair, we measured the chronic and acute sensitivity of the RNase T knockout *E. coli* strain (Δ*rnt*)?against various DNA-damaging agents, including hydrogen peroxide (H_2_O_2_), methyl methanesulfonate (MMS), 4-nitroquinoline-1-oxide (4NQO), mitomycin C (MMC), and UV light. A number of exonucleases that have been shown to play a role in DNA repair, including ExoI (Δ*exoI*) [7],[10], ExoX (Δ*exoX*) [7], PNPase (Δ*pnp*) [29], and RecJ (Δ*recj*) [30], were tested in parallel for a comparison (Table 1 and Figures S1 and S2). The wild-type K-12 strain resisted all DNA-damaging agents when present at a chronic dose, whereas RNase T-deficient strain (Δ*rnt*) had a slow growth phenotype and was sensitive to the chronic dose of H_2_O_2_, MMS, 4NQO, and UV-C (Figure 1A). The RNase T-deficient strain (Δ*rnt*) was also sensitive to the acute dose of H_2_O_2_ in various concentrations from 20 to 80 mM (Figure 1B). The sensitivity of Δ*rnt* strain to UV-C was different from those observed in the previous report [21]; therefore, we further confirmed the UV and H_2_O_2_ sensitivity by *rnt*-rescued experiments, which restored the resistance of Δ*rnt* cells against UV-C and H_2_O_2_ (see Figure S1). This result shows that the sensitivity of the RNase T knockout cells to UV-C and H_2_O_2_ is indeed due to the deficiency of RNase T.

**Table 1:** Summary of the sensitivity of exonuclease knockout *E. coli* k-12 strains against various DNA-damaging agents.

	Acute Sensitivity					Chronic Sensitivity				
		H_2_O_2_ [6]				H_2_O_2_	MMS	4NQO	MMC	UV-C
E. coli strains	Control	20	40	80	Control	1 mM	3 mM	5 µM	120 nM	20 J/m^2^
Wild-type	−	−	−	−	−	−	−	−	−	−
Δrnt (RNase T)	−	−	+	+	−	+	+	+	−	+
Δsbcb (ExoI)	−	−	−	−	−	+	−	−	−	−
ΔexoX (ExoX)	−	−	−	−	−	−	−	−	−	−
Δpnp (PNPase)	−	−	+	+	−	+	+	−	−	+
ΔrecJ (RecJ)	−	−	−	−	−	−	−	+	−	+

H_2_O_2_ produces a wide variety of DNA lesions, including single-strand/double-strand breaks (DSB), oxidation and deamination of bases, and sugar modifications [31],[32], that are usually restored by direct repair (DR), base excision repair (BER), and alternative repair (AR) [1],[29],[30],[33]. DNA alkylating agent MMS produces methylated DNA bases that can be restored by DR and BER [34],[35]. MMS also leads to the accumulation of single-strand gaps (SSGs) and DSB-related DNA damage [29],[35]. MMC is a DNA cross-linking agent that can trigger the SOS response and creates damage repaired by NER [29],[36],[37]. UV light-mimetic agent 4NQO can produce replication-blocked DNA base adducts, SSGs, and DSB-related DNA damages [29],[38] that are mainly repaired by NER [38]. UV-C irradiation (100–290 nm) leads to three major base modifications and DSB-related DNA damage that are usually repaired by BER and DNA recombination [31],[39],[40]. The sensitivity of the Δ*rnt* strain to H_2_O_2_, MMS, 4NQO, and UV-C suggests that RNase T may play a role in BER, AR, and DSB-related DNA repair pathways.

In comparison to the known DNA-repair exonucleases, RNase T had a wider sensitivity to various DNA-damaging agents. The ExoI-deficient cells were only sensitive to H_2_O_2_; the ExoX-deficient cells were not sensitive to any DNA-damaging agents; the PNPase-deficient cells were sensitive to H_2_O_2_, MMS, and UV-C; and the RecJ-deficient cells were sensitive to 4NQO and UV-C (Table 1 and Figure S1). RNase T had a more apparent and wider sensitivity as compared to those of ExoI, ExoX, PNPase, and RecJ, suggesting that RNase T plays more extensive and crucial roles in various DNA repair pathways.

To further characterize the role of RNase T in DNA repair pathways, the single-stranded DNA containing a methylated, deaminated, or oxidized base, or an abasic site at the 3′-terminal end (5′-GAGTCCTATA**X**-3′) were incubated with RNase T in the DNA digestion experiment. We found that RNase T digested the DNA with a methylated base—O^4^-methylthymine (O^4^-mT) and O^6^-methylguanine (O^6^-mG)—and a deaminated base—uracil and hypoxanthine. However, the DNA with a 3′-terminal oxidized base, 8-oxoguanine (8-oxo), and an abasic site were more resistant to RNase T digestion (see Figure 1C). This result suggests that RNase T can function as an exonuclease in the excision step for methylated and deaminated bases in BER and AR.

### RNase T Processes Bulge and Bubble DNA Without Sequence Preference

**Figure 2:**
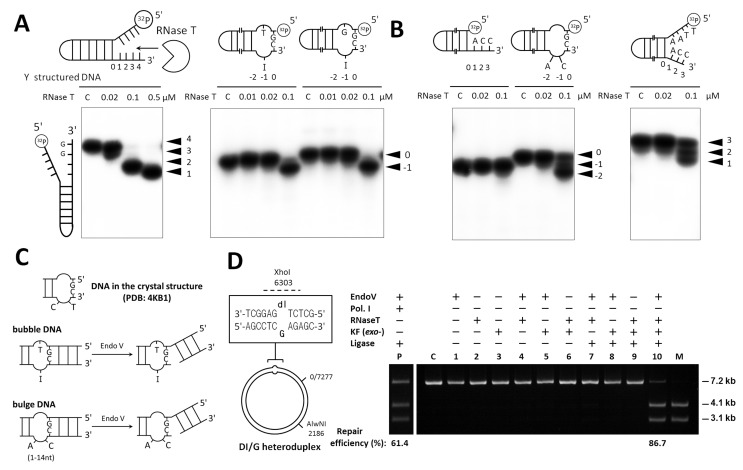
RNase T digests Y-structured, bubble, and bulge DNA and works with Endo V in DNA repair. (A) RNase T digested Y-structured DNA and inosine-containing bubble DNA (sequences listed in Table S1). (B) RNase T could not digest a duplex DNA with a 3′-CC overhang. However, RNase T digested a bulge DNA and a Y-structured DNA with a 3′-CC sequence. The 3′ tails are numbered and the corresponding cleavages are indicated by arrowheads on the right of the gel. (C) Endo V–dependent DNA repair is initiated by Endo V cleavage at the 3′ side of the second phosphodiester bond to the DNA lesions, such as deoxyriboinosine and insertion/deletion DNA. The mispaired I-T site has a bubble DNA structure, whereas the insertion/deletion DNA has a bulge DNA structure. (D) RNase T worked with Endo V to restore an inosine to cytosine. The heteroduplex DNA plasmid contained a mispaired I-G that could be cleaved by XhoI and AlwNI into two linear 4.1- and 3.1-kb fragments if I-G was repaired to C-G. This heteroduplex plasmid was repaired more efficiently (86.7%) following incubation with Endo V, RNase T, KF exo^−^ (the DNA polymerase I Klenow fragment with defective exonuclease activity), and ligase than incubation with Endo V, DNA polymerase I, and ligase (61.4%).

The next question we tackled was what types of DNA that can be processed by RNase T in DNA repair, besides the single-stranded DNA with a lesion. RNase T is not an appropriate exonuclease for digesting single-stranded DNA since its exonuclease activity is easily blocked by any C within a DNA strand. A variety of intermediate structured DNAs are generated during DNA repair, such as bulge, bubble, and Y-structured DNA. Bulge DNAs are produced in frameshift DNA mutations during DNA replication of repetitive sequences [41], whereas bubble DNA are generated in mismatch replication or deamination of DNA bases [42]. Y-structured DNAs are generated in various DNA repair pathways, such as mismatched DNA repair and DNA recombination (see Discussion). To test if RNase T processes these intermediate structured DNA, we incubated RNase T with different DNA and found that RNase T can digest Y-structured DNA and blunt-end bubble DNA with an I-T or I-G bubble (Figure 2A). In digesting the Y-structure DNA, the exonuclease activity of RNase T was blocked by the duplex structure—that is, double-strand effect—and RNase T generated a final product of 1-nucleotide 3′ overhang duplex (Figure 2A). In digesting bulge and bubble DNA, the double-strand effect did not occur, and the blunt-end bulge and bubble DNA was cleaved by RNase T (Figure 2A).

We further tested the sequence preference of RNase T in digesting the structured DNA. In digesting a classical duplex DNA with a short 3′ overhang, the exonuclease activity of RNase T was blocked by a dinucleotide 3′-end CC sequence (Figure 2B). In contrast to the duplex DNA, the bulge and Y-structured DNA with terminal 3′-CC were processed by RNase T into a final product with a 1-nucleotide 3′ overhang (Figure 2B). Therefore, in digesting bubble and bulge DNA, RNase T has no sequence preference, and it removes the last paired nucleotide of any sequence to generate a 1-nucleotide 3′ overhang. In digesting Y-structured DNA, RNase T also has no sequence preference and processes the 3′ tail of any sequence close to the duplex region to generate a 1-nucleotide 3′ tail as the final product.

### Crystal Structure Reveals How RNase T Binds and Processes Blunt-End Bulge DNA

**Figure 3:**
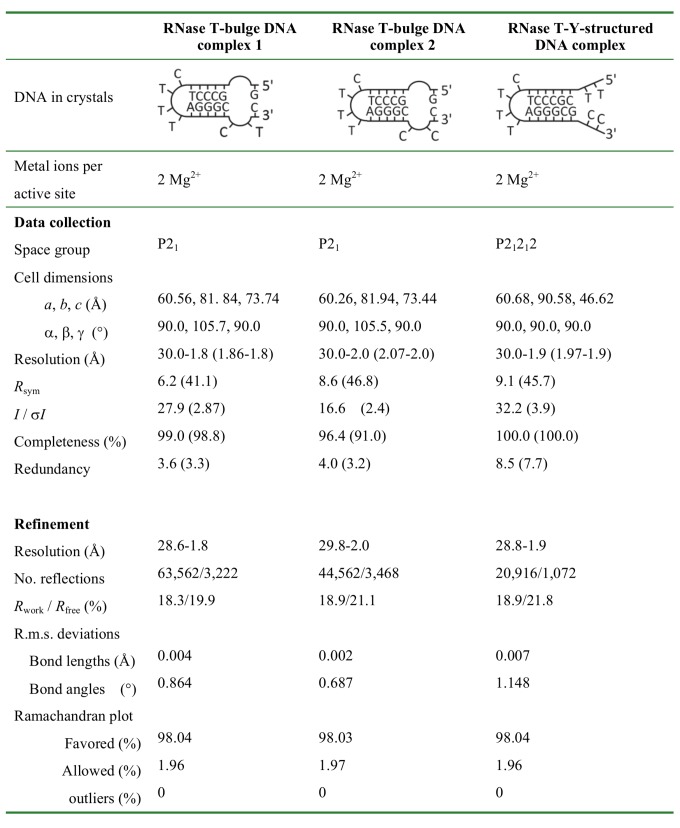
X-ray data collection and refinement statistics for RNase T-DNA complexes.

**Figure 4:**
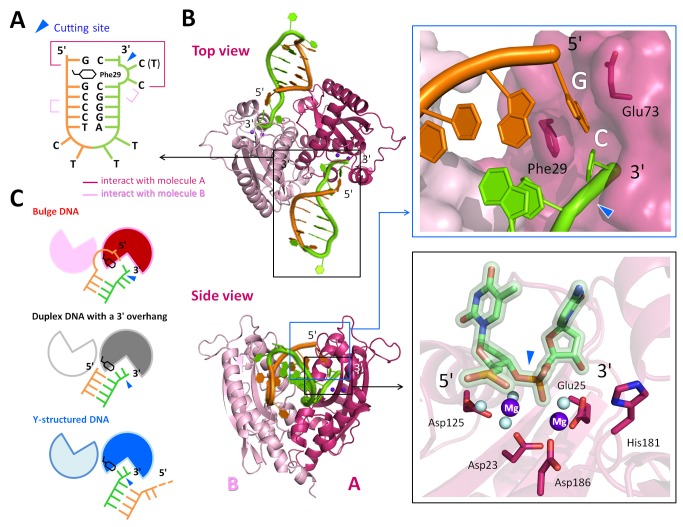
Crystal structures of the RNase T in complex with a bulge DNA. (A) Schematic diagram of the bulge DNA and its interactions to RNase T in the crystal structure (PDB ID code 4KB1). The scissile phosphate cleavage site is indicated by an arrowhead. (B) The top view and side view of the overall crystal structure of the dimeric RNase T in complex with two bulge DNAs. The magnified view in the right top panel shows that Phe29 inserts into the bulge to stack with the neighboring guanine bases. The magnified view in the right bottom panel shows that the active site of RNase T in the complex has an active conformation with two bound magnesium ions. The light blue balls are water molecules. (C) Schematic diagram of three different binding modes for RNase T bound to a bulge DNA (this study, PDB ID codes 4KB0 and 4KB1), a duplex DNA with a short 3′ overhang (previous study [26],[27], PDB ID codes 3NH2 and 3VA3), and a Y-structured DNA (this study, PDB ID code 4KAZ). For clarity, only one of the two DNA molecules bound to RNase T is shown. See Movies S1 and S2.

We were intrigued by how RNase T could bind and process a bubble or bulge in DNA with a blunt end. Previous studies showed that the double-strand effect of RNase T requires a 3′ overhang of a duplex with a length of more than 2 nucleotides for inserting into the active cleft for digestion (see Movie S1). To reveal how RNase T binds and processes a DNA bulge with a blunt end, we co-crystallized RNase T with two bulge DNA molecules, one with a 3′-end TC and one with a 3′-end CC sequence in acidic conditions, pH 5.5 and pH 6.0, respectively (see Table S2). RNase T only digests nucleic acids in basic conditions because the general base H181 has to be deprotonated to activate a nucleophilic water for hydrolysis. Therefore, due to the low pH, the bulge DNA in the crystal were not cleaved by RNase T. The crystal structure of the two complexes was solved by X-ray diffraction methods at a resolution of 1.8 and 2.0 Å, respectively (see Figure 3). In the RNase T–bulge DNA complex structures, the dimeric RNase T bound to two bulge DNAs, with the 3′ end of DNA binding at the active site of each protomer (Figure 4). The bulge DNA was bound between the two RNase T protomers, in a way similar to that of the classical duplex DNA with a 3′ overhang [28].

However, in contrast to the previous duplex DNA complex, the aromatic side chain of Phe29 was inserted into the bulge and stacked with the two neighboring GC base pairs in both of the bulge DNA complexes (see Figure 4A and 4B). In the previous duplex DNA complex, Phe29 was stacked with the 5′-end base of the opposite nonscissile strand, and the stacking stopped the further cleavage of the scissile strand at the 3′ end, resulting in the double-strand effect (see the schematic comparison in Figure 4C). The crystal structure of the bulge DNA complex revealed how RNase T can overcome the double-strand effect by inserting Phe29 into the bulge so that the 3′-end scissile phosphate was moved accordingly into the active site (see Movie S2). We found that the active site of the bulge DNA complex indeed had an active conformation with two bound Mg^2+^ ions, and the general base His181 was located close to the scissile phosphate (Figure 4B).

The crystal structure of the bulge DNA complex also revealed how RNase T could overcome the C effect. The 3′-end cytosine was paired with the 5′-end guanine, and this base pairing prevented Glu73 from interacting with the 3′-end C to induce the C effect (Figure 2B, Figure S2B). Therefore, the bulge DNA could be processed by RNase T without any sequence preference. Moreover, the 5′ end of bulge DNA was not hindered by any residue and could further extend (Figure S2A), suggesting that RNase T can cleave bulge DNA with a long single-stranded region at the opposite strand, similar to those DNA in the frameshift DNA mutations (see Discussion) [41]. The crystal structure thus reveals at the atomic level how RNase T binds and processes a bulge DNA with a blunt end without a sequence preference.

### RNase T Is Likely a Downstream Exonuclease That Follows Endo V Nicking

The bubble and bulge DNA can be produced by Endo V, which makes a nick at the 3′ side one base pair away from a damage site with a deaminated base in the alternative DNA repair [42]. Endo V also processes mismatched DNA, hairpin-containing DNA, bulge DNA, and flap DNA [43]–[45], however the downstream process following Endo V nicking has not been characterized. The bulge DNA in our crystal structures had a conformation similar to the bubble DNA produced by Endo V nicking, suggesting that RNase T might be the downstream exonuclease of Endo V, responsible for removing the last base-paired nucleotide at the 3′ end to release the single-stranded DNA or the damaged DNA bases, such as hypoxanthine, xanthine, and uracil (Figure 2D).

To test this possibility, we prepared the hypoxanthine-containing—that is, inosine-containing—heteroduplex DNA for examination of Endo V–dependent inosine excision repair *in vitro* [46]. The heteroduplex DNA plasmid contained the I-G base pair with an AlwNl cutting site and a potential XhoI cutting site. Once the inosine was restored to cytosine, the plasmid could be cleaved by AlwNl and XhoI into two linear double-stranded DNA molecules of 4.1 and 3.1 kilobases (Figure 2D). The I-G–containing plasmid was then incubated with Endo V, RNase T, ligase, and the Klenow fragment exo^−^ (Polymerase I Klenow fragment with a defected 3′–5′ exonuclease activity). The inosine in the plasmid was restored to cytosine with a higher repair efficiency (86.7%) as compared with those incubated with the wild-type DNA Polymerase I with a proofreading exonuclease domain (61.4%) (Figure 2C). The repair efficiency was positively correlated with the RNase T concentration and the time of incubation (Figure S3). Interestingly, ExoI and ExoX could not work with Endo V to restore the inosine to cytosine (unpublished data). These results show that RNase T can work with Endo V in the Endo V–dependent DNA repair.

### Crystal Structure Reveals How RNase T Processes Y-Structured DNA in a Unique Way

**Figure 5:**
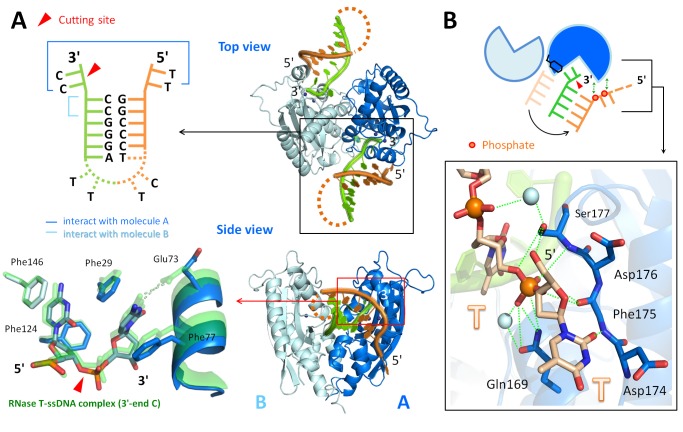
Crystal structures of RNase T in complex with a Y-structured DNA. (A) The overall crystal structure (top and side view) shows that the dimeric RNase T is bound to two Y-structured DNA, with each DNA bound to one protomer. The schematic diagram in the upper left panel shows the Y-structure DNA with a dotted line indicating the disordered DNA region in the crystal structure (PDB ID code 4KAZ). The left bottom panel shows that the 3′ terminal C in the Y-structured DNA (in blue) did not form hydrogen bonds with Glu73 and did not shift and induce inactive conformational changes in the active site as compared to that of a single-stranded DNA with a 3′-C (PDB ID code 3V9Z, in green). (B) Schematic diagram of RNase T in complex with a Y-structured DNA showing that the nonscissile strand rotated about 180° to avoid hindrance by Phe29. The cleavage site is indicated by an arrowhead, and the phosphates in the nonscissile stand making direct interactions with RNase T are shown as red spheres. The close view of the interactions between RNase T and the 5′ overhang of the Y-structured DNA is displayed in the bottom panel. Green dotted lines mark the hydrogen bonds between DNA, water, and RNase T. The light blue balls are water molecules. See Movie S3.

Beside bubble/bulge DNA, RNase T also processed Y-structured DNA, which can be generated during various DNA repair pathways, such as mismatch repair and DNA recombination. However, it remained unknown how an exonuclease can specifically process the 3′-end tail of the intermediate Y-structured DNA. To reveal how RNase T binds and processes a Y-structured DNA, we co-crystallized RNase T with a Y-structured DNA and solved the complex crystal structure at a resolution of 1.9 Å (Figure 3 and Figure 5). In the crystal structure, the Y-structured DNA was bound to RNase T in a unique way, different from those of the bulge DNA and the duplex DNA that were bound between the two protomers with one strand of DNA bound to one protomer (see Figure 4C). In contrast, both strands of the Y-structured DNA were bound to a single protomer, one Y-structured DNA bound to protomer A and the other DNA molecule bound to protomer B (Figure 5). This unique binding mode can avoid the hindrance produced by Phe29, which might stack with the 5′-end base of the opposite nonscissile strand if the Y-structured DNA was bound in a way similar to that of a duplex DNA. Therefore, in this complex, the opposite nonscissile strand of the Y-structured DNA rotated about 180° to interact with the same protomer of RNase T (see Figure 5B). Several residues, including Gln169, Asp174, Phe175, and Ser177, interacted with the nonscissile strand forming hydrogen bonds with the first and second phosphates in the 5′-overhang region, making it fit snugly onto the molecular surface of RNase T (Figure S5).

The 3′ tail of the Y-structured DNA in the crystal structure had a dinucleotide 3′-CC sequence. However, the 3′-CC did not induce the C effect and inhibit the exonuclease activity of RNase T. A close look at the crystal structure of the Y-structured DNA complex showed that the 3′-end C did not interact with Glu73 as it did in the duplex complexes (left panel in Figure S4B). Moreover, the scissile phosphate of the 3′-end C did not shift away from the active site, and as a result, two Mg^2+^ ions were bound in the active site in an active conformation (right panel in Figure S4B). Therefore, due to the unique binding mode, the C effect did not occur when RNase T was bound to a Y-structured DNA with a 3′-end CC. In summary, this crystal structure reveals how RNase T binds a Y-structured DNA in a unique way and how it processes the 3′ tail of any sequence close to the duplex region (see Movie S3).

### RNase T Rather Than ExoI and ExoX Trims Structured DNA Near the Duplex Region

Besides RNase T, two monomeric DnaQ-like exonucleases ExoI and ExoX also process DNA during DNA repair in *E. coli*. ExoI is suggested to play a role in BER [47], mismatch repair [7],[10],[48],[49], UV-related repair [41],[50], and DNA replication [51]. ExoX is involved in mismatch repair [11],[12] and UV-related repair [8]. ExoI binds and cleaves long single-stranded DNA [52], whereas ExoX digests both single-stranded and double-stranded DNA [8]. The exonuclease activity of RNase T, ExoI, and ExoX probably overlap and are redundant in these pathways or they may target different substrates. To compare the substrate preference of RNase T to those of ExoI and ExoX, we further expressed and purified ExoI and ExoX for DNA digestion assays. The dynamic light scattering confirmed that ExoI and ExoX were monomeric proteins in contrast to RNase T, which existed as dimeric proteins (Figure S5).

**Figure 6:**
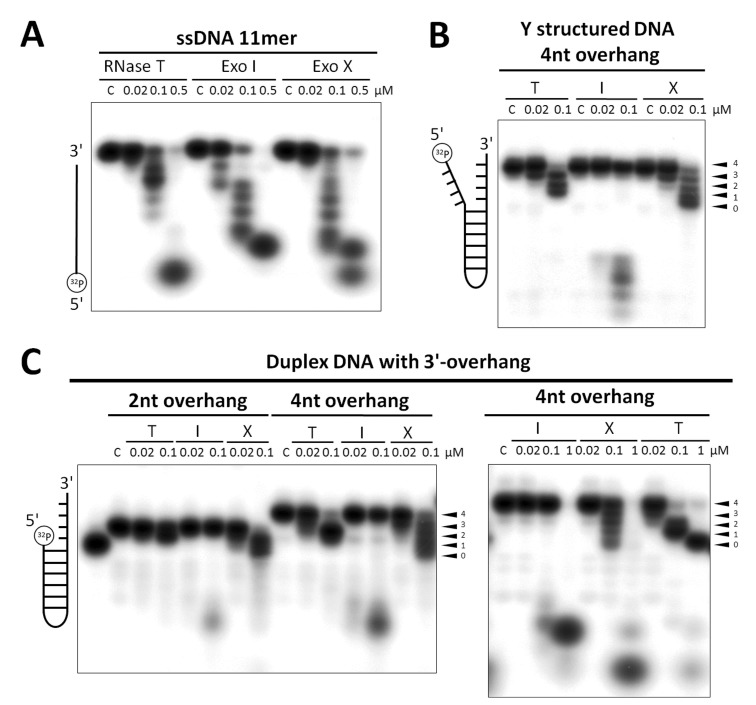
The DNA substrate preference comparison between RNase T, ExoI, and ExoX. (A) The 5′-end ^32^P-labelled single-stranded 11-nucleotide DNA molecules were digested with similar efficiencies by RNase T, ExoI, and ExoX. (B) RNase T is more specific than ExoX in digesting a Y-structured DNA and generated the end product with a 1-nucleotide overhang. ExoI did not digest a Y-structured DNA at low enzyme concentrations. (C) RNase T is more specific than ExoX at digesting a duplex DNA with a short overhang to generate a more specific end product of a duplex with a 1-nucleotide overhang. ExoI did not digest duplex DNA with a short overhang at low enzyme concentrations.

We found that RNase T, ExoI, and ExoX digested single-stranded 11-nucleotide DNA with similar efficiencies (Figure 6A). However, in digesting Y-structured DNA with a short 3′ overhang, only RNase T and ExoX could process the 3′ overhang close to the duplex region, whereas ExoI did not digest Y-structured DNA at low concentrations but did digest Y-structured DNA randomly into small nucleotides at high concentrations (Figure 6B). In digesting duplex DNA with a short 3′ overhang, RNase T processed DNA into a specific length close to the duplex region, generating a final duplex product with a 1-nt 3′ overhang at low concentrations (Figure 6C). ExoX also digested duplex substrates but was less specific, generating various end products with 3′ overhangs of different lengths. On the contrary, ExoI could not digest the duplex substrates at the low concentration (0.02 µM) (Figure 6C). At the high exonuclease concentrations (0.1 and 1 µM), both ExoI and ExoX digested the duplex DNA substrates in the single-stranded and double-stranded regions into small nucleotides. However, RNase T still retained its specificity, only cleaving in the 3′ overhang but not in the duplex region (Figure 6C). These results suggest that RNase T is a highly specific exonuclease that targets the 3′ overhang of structured DNA and produces a precise final product. On the other hand, ExoX is less specific and generates 3′ overhangs of different lengths in digesting duplex substrates with 3′ overhangs, whereas ExoI is specific for single-stranded DNA.

Besides DNA digestion assays, the gel shift assays further showed that RNase T bound with similar affinities to single-stranded DNA, duplex DNA with 4-, 6-, and 10-nucleotide 3′ overhangs (Figure S6). In contrast, ExoI had lower binding affinity for duplex DNA with short 3′ overhangs, such as 4 and 6 nucleotides, in agreement with its low activity for these substrates. ExoX also preferred to bind to single-stranded DNA, but not duplex DNA with short 3′ overhangs at similar concentrations (Figure S6). Combining these results of the exonuclease activity and DNA-binding assays, we conclude that RNase T is the ideal exonuclease for trimming the 3′ overhang of structured DNA closely to the duplex region, including Y-structured DNA and duplex DNA, whereas ExoI and ExoX mainly process single-stranded DNA in DNA repair.

## Discussion

### RNase T Digests Structured DNA in Endo V–Dependent DNA Repair

Our results suggest that RNase T is likely involved in the Endo V–dependent DNA repair pathway. Endo V is a conserved endonuclease playing critical roles in maintaining genome stability in prokaryotes and eukaryotes [53]. Endo V recognizes bubble DNA with mismatched base pairs and deaminated DNA lesions and initiates the Endo V–dependent DNA repair pathway that is independent of BER and MMR [42],[44],[45],[53],[54]. Moreover, Endo V nicks frameshift and structured DNA, such as insertion/deletion loops, hairpins, and flap DNA [43],[55]. Frameshift DNA mutations are mistakenly generated during replication of repetitive sequences [41], and as a result, the bulge DNA are produced by slipped misalignment of tandem repeats [56],[57]. Rearrangements between tandem repeated DNA are important factors for genome instability and have been implicated in Friedreich ataxia in humans [58],[59]. Slipped misalignment of tandem repeat DNA may cause palindrome-stimulated deletion or expansion by two RecA-independent recombination mechanisms—that is, single-strand annealing and replication slipped mispairing [60],[61]. Single-strand-specific exonucleases, such as ExoI, ExoX, and RecJ, were reported to stabilize tandem repeats and limit RecA-independent recombination [56],[62]. However, the downstream structure-specific exonuclease of Endo V for the further trimming of the DNA from the broken end has not yet been identified.

**Figure 7:**
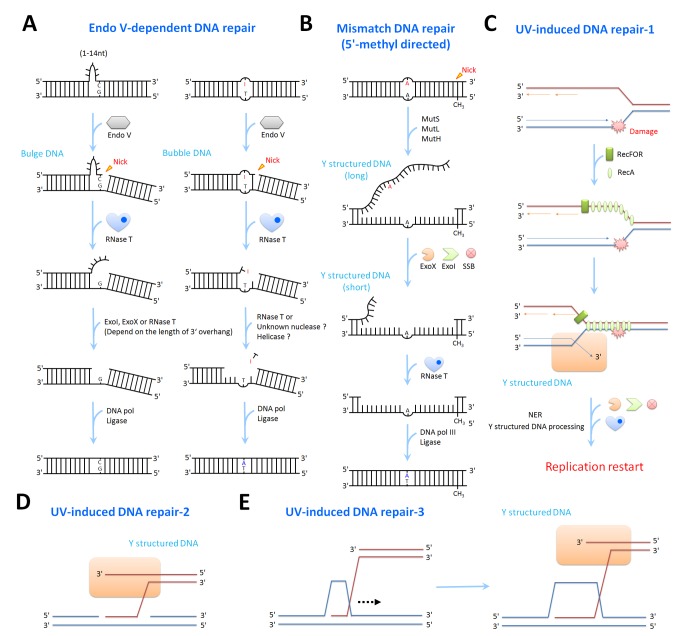
Possible roles of RNase T in Endo Vdependent, mismatch, and UV-induced DNA repair. &lparA)Endo V makes a nick at the 3′ side one base pair away from the damaged sites, including insertion loops and deaminated sites. RNase T further trims the bulge and bubble DNA and removes the last base-paired structure to release the single-stranded DNA insertions or damaged DNA base for the next-step processing. ExoI, ExoX, and RNase T further trim the flapped 3′ overhang, followed by DNA polymerase and ligase activity to complete the repair pathway. (B) In the mismatch DNA repair pathway, a Y-structured DNA is produced after processing by MutS, MutL, and MutH. It is likely that ExoI and ExoX trim the long 3′ tail with the help of SSB and RNase T is responsible for the final trimming of the short 3′ overhang of the Y-structured DNA. (C) In the UV-induced DNA repair by replication restart pathway, the damaged DNA are bound and annealed by RecA and RecFOR. As a result, the Y-structured DNA (in the orange box) is generated that can be further digested by RNase T. (D) The Y-structured DNA can be generated in gap-filling homologous recombination and (E) RecA-dependent homologous recombination. RNase T may remove the short 3′ overhang of these Y-structured DNA in these UV-induced DNA repair pathways.

Our structural and biochemical data of RNase T show that it can bind and digest bulge/bubble and Y-structured DNA. Moreover, RNase T can work with Endo V, DNA Polymerase I (Klenow fragment exo^−^), and ligase to restore an inosine to cytosine in a heteroduplex DNA molecule *in vitro*. The crystal structures of RNase T bound with a blunt-end bulge DNA further show how RNase T removes the last base pair at the 3′ end by a special Phe-inserting binding mode. All these results suggest that RNase T may function as the downstream exonuclease of Endo V in alternative DNA repair. Taking together these lines of evidence, we suggest that RNase T likely recognizes these bulge and bubble DNA structures generated by Endo V and trims at the 3′ end of the nicked site to remove the last base pair next to the lesion. The single-stranded DNA or damaged DNA is then released for the next step of processing (see Figure 7A).

After removing the 3′-end base-paired nucleotide by RNase T, insertion DNA, hairpin DNA, and deaminated DNA lesions are released as single-stranded DNA. These single-stranded insertion DNA and hairpin DNA are probably further trimmed by the single-strand-specific exonucleases, such as ExoI and/or ExoX, with the help of single strand binding protein (SSB) and helicases. RNase T can further digest the 3′-end short overhang close to the duplex region in a way that we observed in the crystal structure of the Y-structured DNA complex. Deaminated DNA lesions are likely also removed by RNase T since we show that RNase T can digest single-stranded DNA containing oxidized bases and deaminated bases (Figure 1C). It has been shown that the dimeric Exo I from *Thermus thermophilus* shares a sequence homology to RNase T and plays a similar role in digesting damaged DNA with methylated and deaminated bases [30]. It is very likely that Exo I from *Thermus thermophilus* is a functional homologue of RNase T and both of them play key roles in DNA repair. Therefore, after nicking by Endo V, the single-strand-specific exonucleases and structure-specific RNase T likely work together to further trim DNA from the broken end. After this trimming, polymerases and ligases can complete the DNA repair pathway.

### RNase T Digests Y-Structured DNA in Various DNA Repair Pathways

RNase T plays crucial roles in various DNA repair pathways, as shown by the sensitivity of the *rnt* knockout strain to a wide range of DNA-damaging agents. The indispensable role of RNase T might be due to its unique specificity for structured DNA that are generated during various DNA repair pathways. For instance, UV radiation can lead to single/double-strand breaks and base modifications, such as cross-linked pyrimidine dimers, photoproducts, and thymine glycols, and as a result, three different UV-induced DNA repair pathways are initiated [39],[63]. In the first pathway, the base modification induced by UV may stall replication forks. In such a case, RecFOR and RecA bind to the lagging strand template and the invasion-containing leading strand to promote double-strand formation and repair by NER [64]. During this process, the Y-structured DNA formed on the leading strand requires a structure-specific exonuclease, very likely RNase T, to trim its 3′ overhang (see Figure 7C).

In the second pathway, UV radiation can induce single-strand breaks that can be repaired by homologous recombination [65]. During this process, Y-structured DNA is formed as an intermediate during gap-filling recombination (see Figure 7D). ExoI was reported to promote this RecA-dependent 5′-end strand exchange by digesting the 3′ competitor strand [66],[67]. However, ExoI cannot digest the 3′ overhang close to the duplex region, and thus most likely RNase T is responsible for processing the Y-structured DNA intermediates in the gap-filling recombination pathway.

In the third pathway, the double-strand breaks induced by UV radiation are generally repaired by the RecA-dependent homologous recombination in bacteria [68]. This DNA repair pathway is initiated by RecBCD or RecJ to generate 3′ overhangs and is followed by RecA and RecFOR to promote strand invasion. DNA repair synthesis is then primed by PolI and PolIII from the invaded strand of the D-loop structure. RuvC resolvase cleaves the Holliday junctions that are synthesized after branch migration and LigA seals the nick to complete the homologous recombination [1]. In this process, ExoI was reported to affect RecBCD-mediated recombination [69] since the 3′–5′ exonucleases are required to degrade the 3′ tail of the intermediate Y-structured DNA after RecA dissociation [48],[51],[70],[71]. Yet ExoI is not an appropriate exonuclease for digesting the 3′ tail near the duplex region. Based on our results, we suggest that most likely RNase T is involved in digesting the 3′ tail close to the duplex region in the UV-induced DNA homologous recombination (Figure 7E). Moreover, in comparison with FEN1, which is a flap endonuclease that binds DNA with one 3′-flap nucleotide and cleaves one nucleotide into the double-stranded DNA at the 5′ flap end to produce a ligatable product during DNA replication and repair [72], RNase T is likely required to produce a DNA with a short 3′ overhang with one or two nucleotides that can be further processed in DNA homologous recombination.

Besides UV-induced DNA repair, RNase T may also participate in other DNA repair processes that require a structure-specific 3′–5′ exonuclease, such as MMR. It has been shown that ExoI and ExoX are essential for methyl-directed mismatch repair in *E. coli* [7],[10]–[12],[49],[50]. These two monomeric exonucleases are responsible for removing the 3′ single-stranded tail in Y-structured DNA during MMR (see Figure 7B). However, they cannot process the 3′ single-stranded tail close to the double-stranded region [1],[49]. ExoI only processes DNA with a long single-stranded region (over 13 nucleotides) in a processive manner, while a SSB stimulates its exonuclease activity [52],[73],[74]. ExoX, however, interacts with MutL during MMR and is not specific for processing Y-structured DNA [8],[12]. On the other hand, the RNase T homolog *Thermus thermophilus* ExoI is suggested to excise the 3′ overhang of a Y-structured DNA and plays a role in MMR [30]. Therefore, it is very likely that RNase T processes the 3′ tail of the Y-structured DNA in MMR in *E. coli.* Our structure and biochemical assays show that the C effect does not occur when RNase T digests short 3′ overhang of a Y-structured DNA, and hence RNase T is capable of processing any sequence of the 3′ overhang of a Y-structured DNA during MMR. Therefore, we propose here that the monomeric ExoI and ExoX work with a helicase or SSB to process long 3′ tails, while the dimeric RNase T further trims the short 3′ overhang of Y-structured DNA during MMR.

In conclusion, RNase T is a unique structure-specific exonuclease responsible for processing the 3′ ends of structured DNA in various DNA repair pathways. RNase T has an ideal dimeric architecture for binding and processing the 3′ end of various structured DNA in diverse ways, including duplex, bulge/bubble, and Y-structured DNA. Therefore, this intriguing exonuclease has multiple functions not only for processing duplex RNA during RNA maturation, but also processing bubble/bulge and Y-structured DNA during DNA repair. The diverse functions and different specificities of RNase T are closely correlated to its dimerization architecture and various binding modes against different substrates. We provide solid data here showing how the dimeric RNase T processes structured DNA in DNA repair that will serve as a model for understanding the molecular functions of thousands of members of DnaQ-like exonucleases.

## Materials and Methods

### Bacteria Strains and Survival Studies

Wild-type *E. coli* K-12, single gene knockout (*Δrnt*, *Δsbcb*, *Δexox*, *Δpnp*, and *ΔrecJ*) strains used in the survival studies were from the Keio collection [75]. All *E. coli* cells were grown to an OD_600_ of 0.5–0.6 in LB medium at 37°C. To measure the acute sensitivity to hydrogen peroxide (H_2_O_2_), cells were exposed to 0, 20, 40, and 80 mM H_2_O_2_ for 20 min. After removing H_2_O_2_, cells were diluted 100-fold into 10 ml LB medium and further grown on a rotary shaker (200 r.p.m.) at 37°C for the measurement of *A_600_* (OD) at 60 min intervals. To measure the chronic sensitivity to H_2_O_2_, MMS, mitomycin (MMC), and 4NQO, serial dilutions of cells were spotted on plates containing indicated concentrations of the DNA-damaging agents and incubated overnight at 37°C. To measure the sensitivity against UV-C, serial dilutions of cells were spotted on plates and exposed to UV-C (254 nm) in 20 J/m^2^ for 10 s by Hoefer UVC 500*-*Ultraviolet Crosslinker (Hoefer Inc.). After UV-C irradiation, cells were incubated overnight at 37°C.

### Protein Expression and Purification

The full-length *rnt*, *sbcb*, and *exox* genes were amplified by PCR using *E. coli* genomic DNA from JM109 or K-12 strains and cloned into NdeI/XhoI sites of expression vectors pET-28a (Novagen) to generate the N-terminal His-tagged fused recombinant proteins. The expression plasmid was transformed into the *E. coli* BL21-CodonPlus(DE3)-RIPL strain (Stratagene) cultured in LB medium supplemented with 35 µg/ml kanamycin. Cells were grown to an OD_600_ of 0.5–0.6 at 37°C and induced by 0.8 mM IPTG at 18°C for 18 h. The harvested cells were dissolved in 50 mM Tris-HCl (pH 7.5) buffer containing 300 mM NaCl and disrupted by a microfluidizer. Each exonuclease was purified by chromatographic methods using a HiTrap TALON column (GE Healthcare), a HiTrap Heparin column (GE Healthcare), and a gel filtration column (Superdex 75, GE Healthcare). Purified RNase T, ExoI, and ExoX samples were concentrated to 15–35 mg/ml in 300 mM NaCl and 50 mM Tris-HCl (pH 7.0).

### DNA Digestion and Binding Assays

DNA oligonucleotides used for nuclease activity assays were synthesized (BEX Co., Tokyo, Japan or MDBio, Inc., Taiwan) and labeled at the 5′ end with [γ-^32^P]ATP by T4 polynucleotide kinase and purified on a Microspin G-25 column (GE Healthcare) to remove the nonincorporated nucleotides. Purified substrates (20 nM; see Table S1 for sequences) were incubated with RNase T, ExoI, or ExoX at various concentrations in a buffered solution of 120 mM NaCl, 2 mM MgCl_2_, and 50 mM Tris-HCl (pH 7.0) at room temperature for 20–60 min. The reaction was quenched by addition of the stop solution (2× TBE) and heating at 95°C for 5 min. Reaction samples were then resolved on 20% denaturing polyacrylamide gels and visualized by autoradiography (Fujifilm, FLA-5000).

DNA binding affinities of RNase T, ExoI, and ExoX were measured by gel shift assays. The 5′-end ^32^P-labeled DNA substrates (20 nM) were incubated with RNase T, ExoI, or ExoX in a solution of 100 mM NaCl, 30 mM EDTA, 10 mM EGTA, and 50 mM Tris-HCl (pH 7.0) for 20 min at room temperature. The concentrations of each protein used in the assays were 0, 5, and 50 µM. Reaction samples were then resolved on 20% TBE gels (Invitrogen) and visualized by autoradiography (Fujifilm, FLA-5000).

### *In Vitro* Endo V–Dependent DNA Repair Assay

The *E. coli* strain NM522.RS5033 was used in the assay as described in Fang et al. [76]. DNA polymerase I (*E. coli*), the Klenow fragment exo^−^ (DNA polymerase I Klenow fragment without the 3′–5′ exonuclease activity), *E. coli* DNA ligase, T4 polynucleotide kinase, recombinant Endo V, and restriction endonucleases were obtained from New England Biolabs. RecBCD nuclease was purchased from EPICENTRE Biotechnologies.

Construction of dI-G heteroduplex DNA substrates was prepared as described in Lee et al. [46]. M13mp18 replicative form DNA was hydrolyzed with HindIII and mixed with a 4-fold molar excess of M13LR1 viral DNA, followed by alkaline denaturation and re-annealing. The excess ssDNA was removed by hydroxyapatite (Biorad) chromatography and benzoylated naphthylated DEAE cellulose (Sigma) chromatography, and the linear dsDNA was removed by RecBCD nuclease (EPICENTRE) treatment. The resulting circular duplex DNA containing 22-nt gaps was further purified by Vivaspin 20 ultrafiltration (GE Healthcare). A 5′-phosphorylated deoxyinosine-containing 22-bp synthetic oligonucleotides, 5′-AGCTCTIGAGGCTGCTGCTGCT-3′ (Blossom Biotech), was then annealed to the gap and sealed by T4 DNA ligase in the presence of ethidium bromide. The covalently closed dG:I heteroduplex DNA was isolated by CsCl-EtBr density gradient centrifugation.

The repair conditions were modified from Lee et al. [46]. The dI-G heteroduplex substrates (0.1 µg) were incubated with repair enzymes (1.1 nM Endo V, 0.13 µM DNA polymerase I/0.13 µM Klenow fragment *exo-*, and 5 µM RNaseT) for 30 min at 37°C in 15-µl reactions containing 50 mM NaCl, 10 mM Tris-HCl (pH 7.9), 10 mM MgCl_2_, 1 mM dithiothreitol, 50 µg/ml bovine serum albumin, 0.3 mM NAD^+^, and 125 µM of each dNTP. The reactions were terminated by heat inactivation at 75°C for 20 min. The DNA was then analyzed by restriction endonuclease hydrolysis and agarose gel electrophoresis. The gel images were captured by a gel documentation CCD camera (UVP Ltd.) using Viewfinder 3.0, and band intensities were then measured by NIH Image J 1.45s software.

### Crystallization and Crystal Structure Determination

Wild-type RNase T (25–35 mg/ml) in 300 mM NaCl and 50 mM Tris-HCl (pH 7.0) were mixed with different stem-loop DNA substrates in the molar ratio of 1∶1.2. Detailed information for DNA sequences and crystallization conditions of the three structures is given in the Table S2. All crystals were cryo-protected by Paraton-N (Hampton Research, USA) for the data collection at 100 K. X-ray diffraction data were collected using synchrotron radiations at SPXF beamline BL13B1 at NSRRC, Taiwan, or at the BL44XU beamline at SPring-8, Japan. All diffraction data were processed by HKL2000, and the diffraction statistics are listed in Table 1. Structures were solved by the molecular replacement method using the crystal structure of *E. coli* RNase T (PDB ID code 3NGY) as the search model by program MOLREP of CCP4. The models were built by Coot and refined by Phenix.

### Accession Numbers

Structural coordinates and diffraction structure factors have been deposited in the RCSB Protein Data Bank with the PDB ID codes of 4KB0 and 4KB1 for RNase T-bulge DNA complexes and 4KAZ for the RNase T-Y structured-DNA complex.

## Supporting Information

Figure S1 **Effects of UV and DNA-damaging agents on various exonuclease-deficient** ***E. coli*** **K-12 strains.** (A) The ExoI, ExoX, PNPase, and RecJ knockout cells were exposed to UV-C for 10 s or different DNA-damaging agents, such as H_2_O_2_, MMS, 4NQO, and MMC, in a chronic dose. The *rnt* rescue experiments for RNase T knockout cells were performed in parallel by transforming the *rnt*-containing plasmid into RNase T knockout cells, which were then exposed to UV-C for 10 s (Δ*rnt*- *rnt*). The *rnt* rescued the sensitivity of the RNase T knockout cells against UV-C. (B) Growth curves of the exonuclease-deficient cells after exposure to H_2_O_2_ in an acute does. The *rnt* rescue experiments (Δ*rnt*- *rnt*) were performed in parallel showing that *rnt* rescued the sensitivity of the RNase T knockout cells against H_2_O_2_. The *rnt*-containing plasmid was prepared as described previously [27].
(TIF)

Figure S2 **The crystal structure of the RNase T in complex with bulge DNA.** (A) The molecular surface of RNase T shows that the 5′ end of the bulge DNA is not hindered by RNase T and can further extend. (B) The 3′ end of the bulge DNA did not form hydrogen bonds with Glu73 (left panel), in contrast to that of single-stranded DNA with a 3′-end C (right panel, PDB ID code 3V9Z). (C) The active site of RNase T-bulge DNA complex reveals two Mg^2+^ ions in the active conformation (left panel), and therefore, RNase T can digest a bulge DNA with a 3′-C. On the other hand, the active site of RNase T-ssDNA complex has only one Mg^2+^ ion in the inactive conformation, and therefore RNase T cannot digest a ssDNA with a 3′-end C (right panel).
(TIF)

Figure S3 **RNase T promotes Endo V–dependent repair efficiency.** (A) Correlation between the repair efficiency and the concentration of RNaseT. The dI-G heteroduplex substrate was incubated with RNase T and Endo V in the Endo V–dependent repair assay. (B) Time course analysis of deoxyinosine correction in Endo V-RNaseT-Klenow fragment (*exo-*) reconstituted excision repair. The dI-G heteroduplex substrate was incubated with 5 µM RNase T at 37°C for indicated times, and reactions were terminated by heat inactivation at 75°C for 20 min. The standard deviations were estimated from at least three independent reactions.
(TIF)

Figure S4 **The crystal structure of the RNase T-Y-structured DNA complex.** (A) The molecular surface of RNase T shows that the 3′ and 5′ end of the Y-structured DNA fit snugly onto its surface. (B) The 3′ end of the Y-structured DNA did not shift up and fit well with the single-stranded DNA with a 3′-end AA (PDB ID code 3V9X). The active site of the Y-structured DNA complex had two metal ions in an active conformation. Therefore, RNase T can bind and digest a Y-structured DNA without sequence preference.
(TIF)

Figure S5 **Domain structures of several DnaQ-like exonucleases.** (A) Domain structures of RNase T, ExoI, ExoX, TTHB178 (ExoI from *T. thermophiles*), TREX1, and TREX2. (B) The purified recombinant RNase T was a homodimer, whereas ExoI and ExoX were monomers, as analyzed by dynamic light scattering.
(TIF)

Figure S6 **Gel shift assays for RNase T, ExoI, and ExoX.** Substrates for these experiments were single-stranded 11-nucleotide DNA (ssDNA 11) and stem-loop DNA with 0-, 4-, 6-, and 10-nucleotide 3′ overhang (SL_0, SL_4, SL_6, and SL_100). Sequences of these DNAs are listed in Table S1.
(TIF)

Movie S1 **How RNase T processes duplex DNA with a 3′ overhang.**
(MOV)

Movie S2 **How RNase T processes bulge/bubble DNA.**
(MOV)

Movie S3 **How RNase T processes Y-structured DNA.**
(MOV)

Table S1 **Substrates for nuclease activity and binding assays.**
(DOCX)

Table S2 **Crystallization conditions of RNase T-Structured DNA complexes.**
(DOCX)

## Abbreviations

4NQO: 4-nitroquinoline-1-oxide
8-oxo: 8-oxoguanine
AR: alternative repair
BER: base excision repair
DR: direct repair
DSB: double-strand breaks
Endo V: Endonuclease V
H_2_O_2_: hydrogen peroxide
MMC: mitomycin C
MMS: methyl methanesulfonate
NER: nucleotide excision repair
O^4^-mT: O^4^-methylthymine
O^6^-mG: O^6^-methylguanine
SSB: single strand binding protein
SSGs: single-strand gaps
