# Supplementary figures and images for: Structural Insights Into DNA Repair by RNase T—An Exonuclease Processing 3′ End of Structured DNA in Repair Pathways
Source: PLoS Biol. 2014 Mar 4;12(3):e1001803. doi: 10.1371/journal.pbio.1001803 (PMC3942315; doi:10.1371/journal.pbio.1001803)

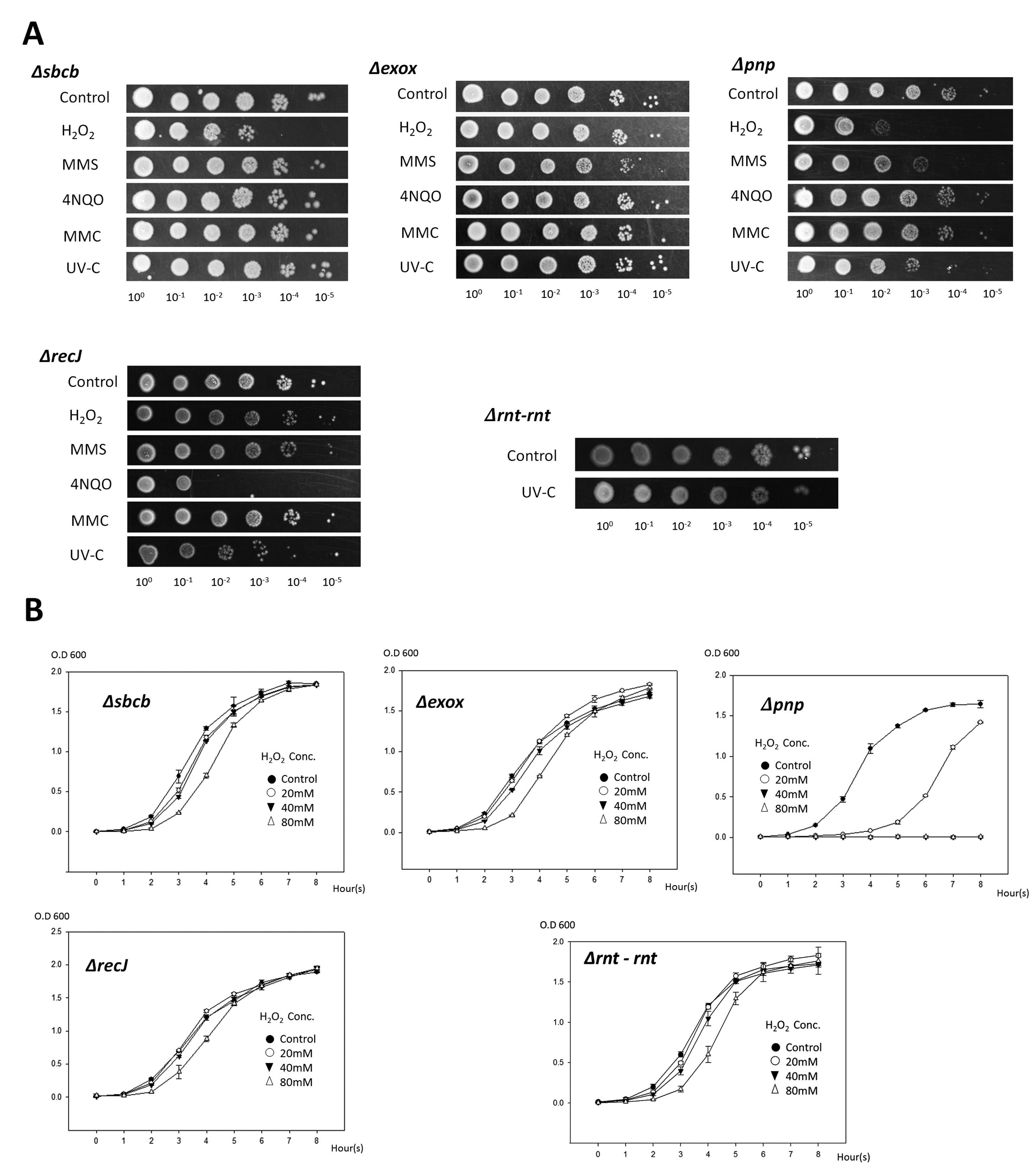

Supplement: Figure S1 — Effects of UV and DNA-damaging agents on various exonuclease-deficient E. coli K-12 strains. (A) The ExoI, ExoX, PNPase, and RecJ knockout cells were exposed to UV-C for 10 s or different DNA-damaging agents, such as H2O2, MMS, 4NQO, and MMC, in a chronic dose. The rnt rescue experiments for RNase T knockout cells were performed in parallel by transforming the rnt-containing plasmid into RNase T knockout cells, which were then exposed to UV-C for 10 s (Δrnt- rnt). The rnt rescued the sensitivity of the RNase T knockout cells against UV-C. (B) Growth curves of the exonuclease-deficient cells after exposure to H2O2 in an acute does. The rnt rescue experiments (Δrnt- rnt) were performed in parallel showing that rnt rescued the sensitivity of the RNase T knockout cells against H2O2. The rnt-containing plasmid was prepared as described previously [27]. (TIF) [file pbio.1001803.s001.tif]

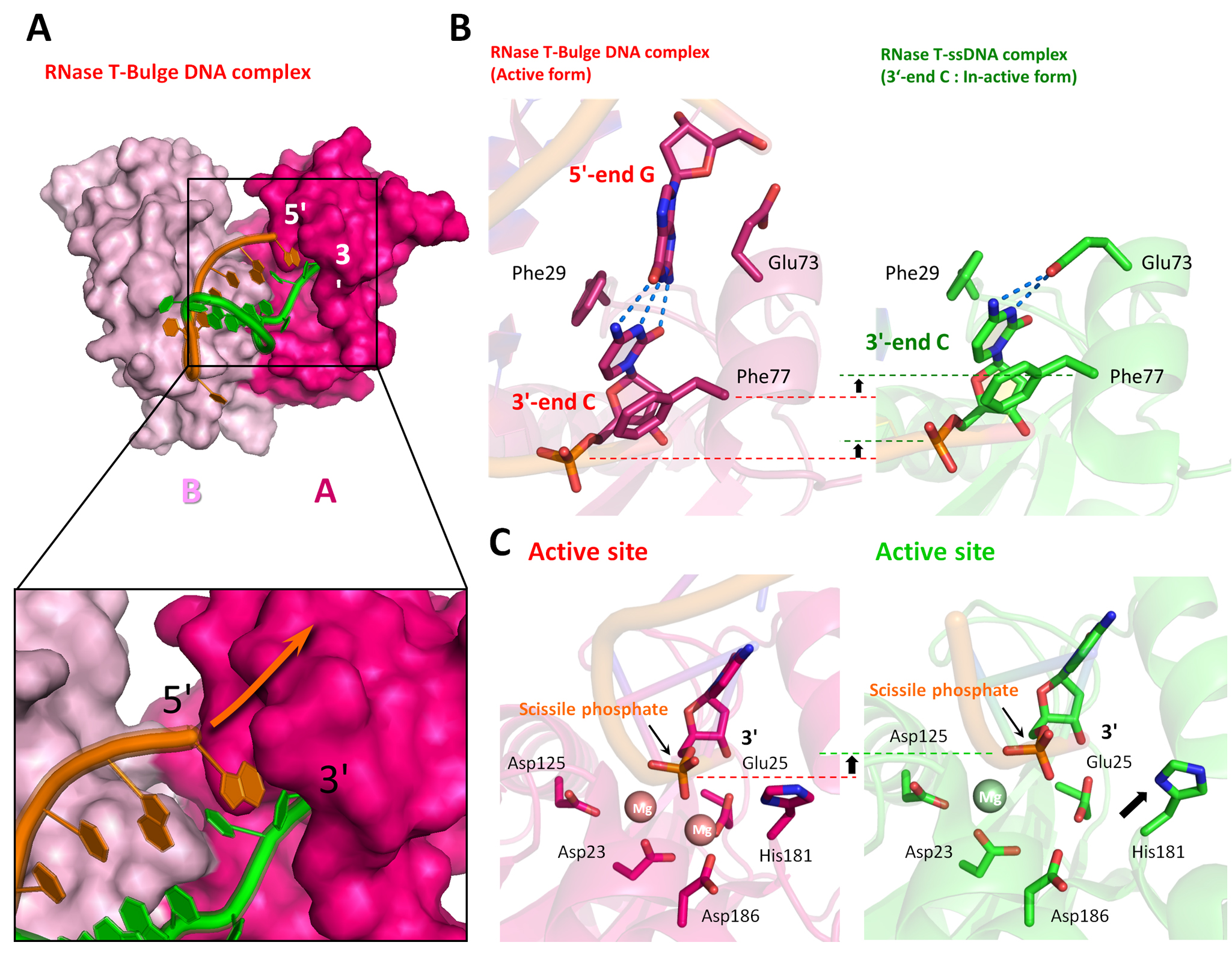

Supplement: Figure S2 — The crystal structure of the RNase T in complex with bulge DNA. (A) The molecular surface of RNase T shows that the 5′ end of the bulge DNA is not hindered by RNase T and can further extend. (B) The 3′ end of the bulge DNA did not form hydrogen bonds with Glu73 (left panel), in contrast to that of single-stranded DNA with a 3′-end C (right panel, PDB ID code 3V9Z). (C) The active site of RNase T-bulge DNA complex reveals two Mg2+ ions in the active conformation (left panel), and therefore, RNase T can digest a bulge DNA with a 3′-C. On the other hand, the active site of RNase T-ssDNA complex has only one Mg2+ ion in the inactive conformation, and therefore RNase T cannot digest a ssDNA with a 3′-end C (right panel). (TIF) [file pbio.1001803.s002.tif]

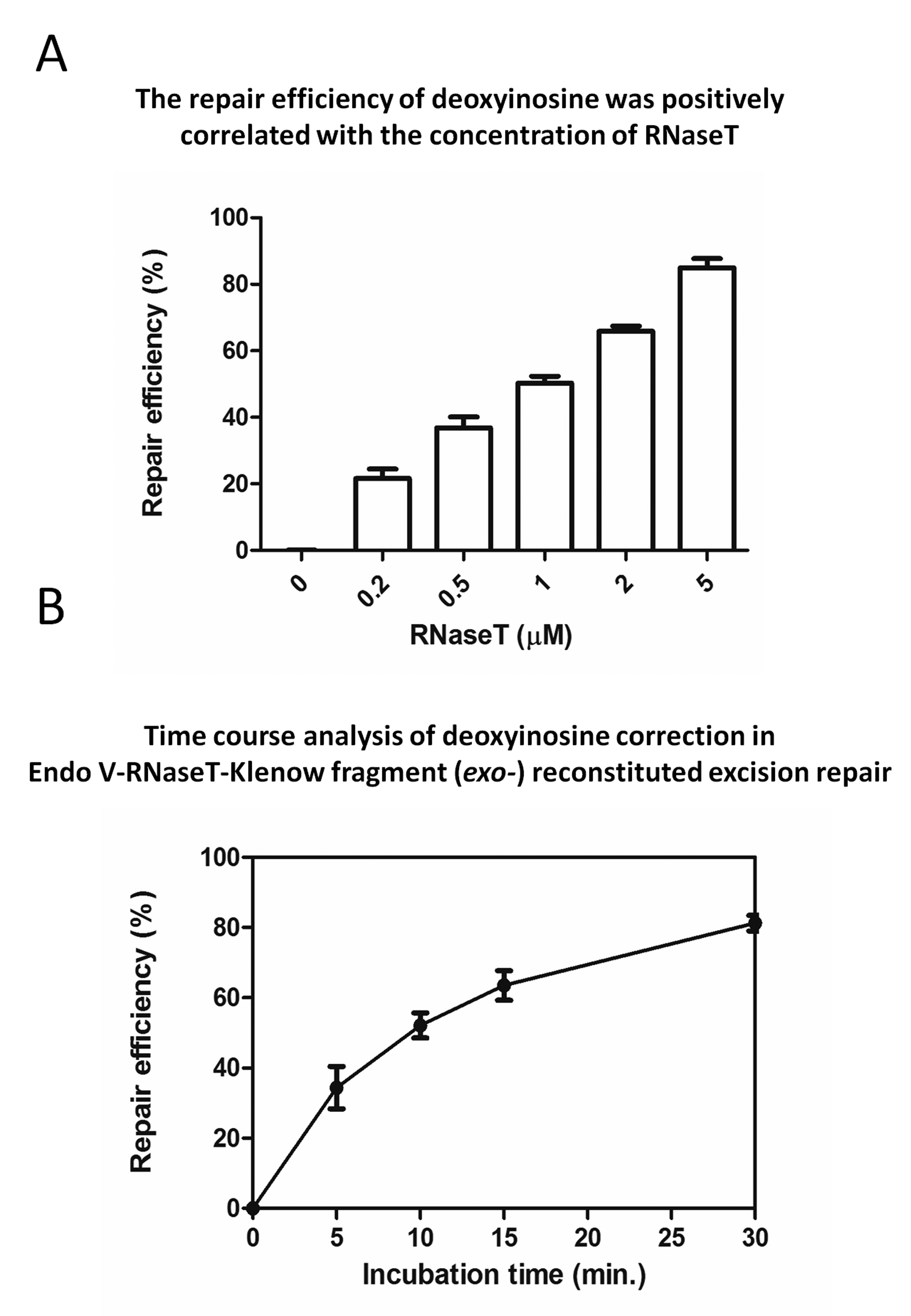

Supplement: Figure S3 — RNase T promotes Endo V–dependent repair efficiency. (A) Correlation between the repair efficiency and the concentration of RNaseT. The dI-G heteroduplex substrate was incubated with RNase T and Endo V in the Endo V–dependent repair assay. (B) Time course analysis of deoxyinosine correction in Endo V-RNaseT-Klenow fragment (exo-) reconstituted excision repair. The dI-G heteroduplex substrate was incubated with 5 µM RNase T at 37°C for indicated times, and reactions were terminated by heat inactivation at 75°C for 20 min. The standard deviations were estimated from at least three independent reactions. (TIF) [file pbio.1001803.s003.tif]

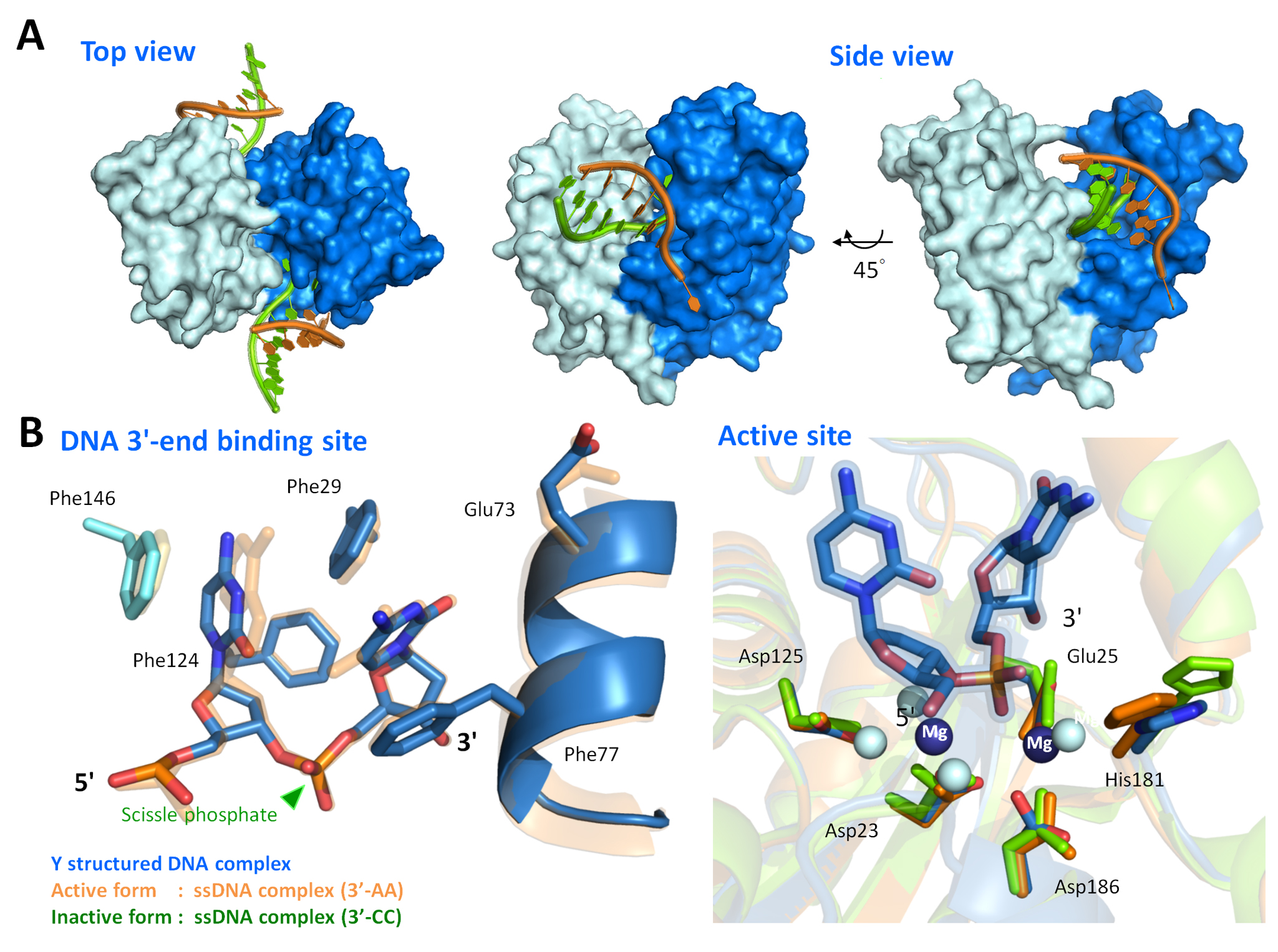

Supplement: Figure S4 — The crystal structure of the RNase T-Y-structured DNA complex. (A) The molecular surface of RNase T shows that the 3′ and 5′ end of the Y-structured DNA fit snugly onto its surface. (B) The 3′ end of the Y-structured DNA did not shift up and fit well with the single-stranded DNA with a 3′-end AA (PDB ID code 3V9X). The active site of the Y-structured DNA complex had two metal ions in an active conformation. Therefore, RNase T can bind and digest a Y-structured DNA without sequence preference. (TIF) [file pbio.1001803.s004.tif]

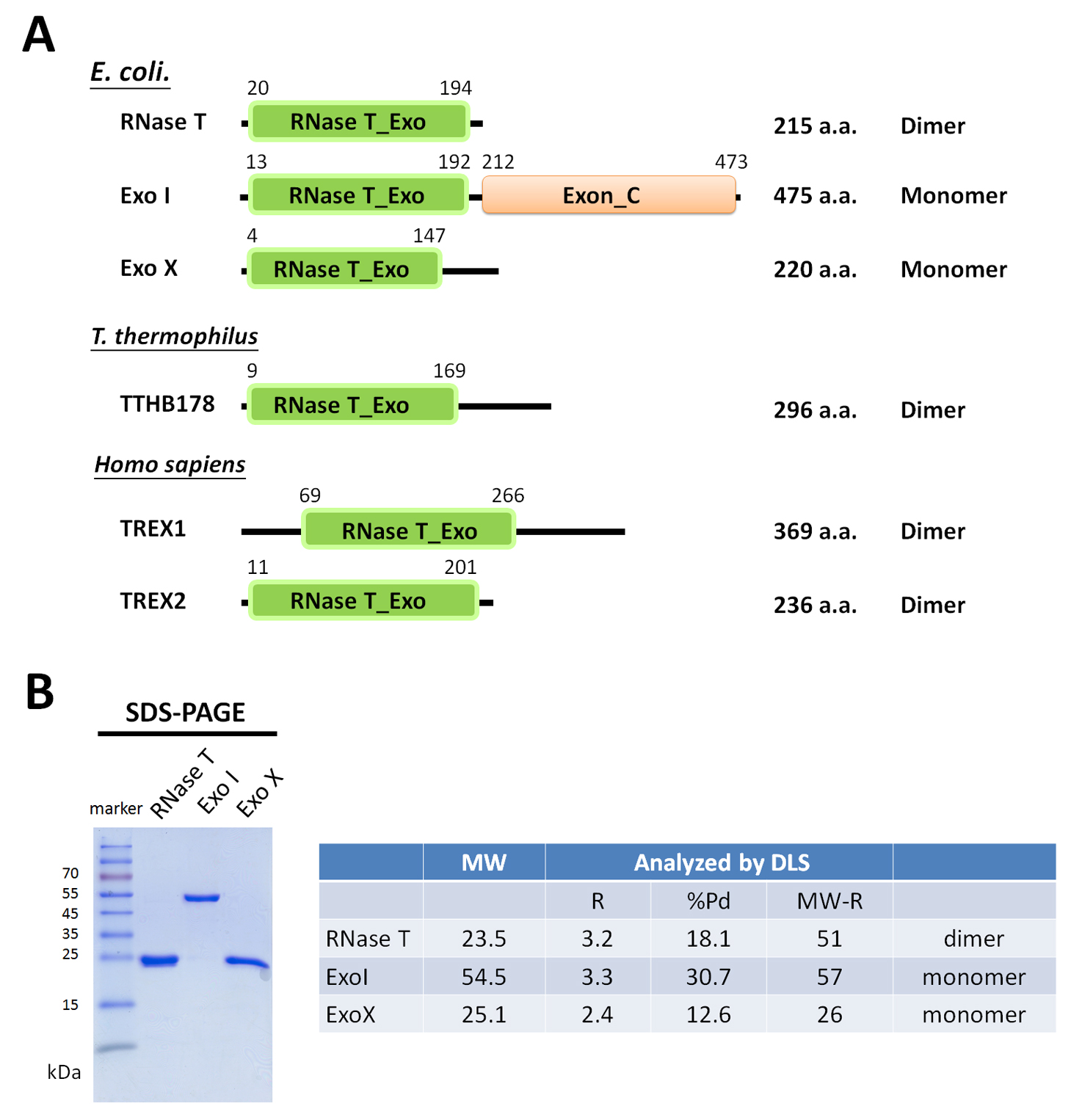

Supplement: Figure S5 — Domain structures of several DnaQ-like exonucleases. (A) Domain structures of RNase T, ExoI, ExoX, TTHB178 (ExoI from T. thermophiles), TREX1, and TREX2. (B) The purified recombinant RNase T was a homodimer, whereas ExoI and ExoX were monomers, as analyzed by dynamic light scattering. (TIF) [file pbio.1001803.s005.tif]

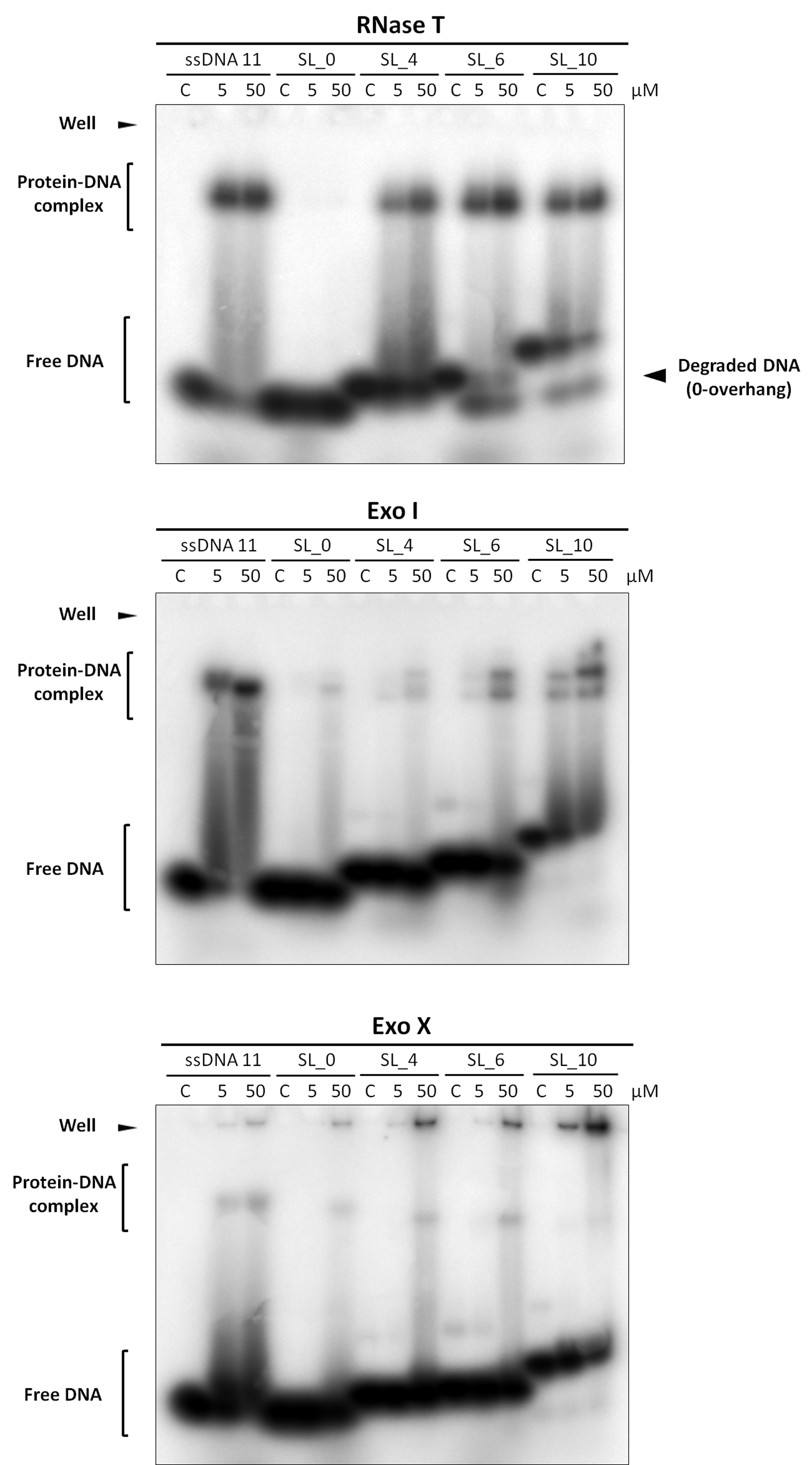

Supplement: Figure S6 — Gel shift assays for RNase T, ExoI, and ExoX. Substrates for these experiments were single-stranded 11-nucleotide DNA (ssDNA 11) and stem-loop DNA with 0-, 4-, 6-, and 10-nucleotide 3′ overhang (SL_0, SL_4, SL_6, and SL_100). Sequences of these DNAs are listed in Table S1. (TIF) [file pbio.1001803.s006.tif]
