# Supplementary material for: Structural Insights Into DNA Repair by RNase T—An Exonuclease Processing 3′ End of Structured DNA in Repair Pathways
Source: PLoS Biol. 2014 Mar 4;12(3):e1001803. doi: 10.1371/journal.pbio.1001803 (PMC3942315; doi:10.1371/journal.pbio.1001803)
Supplement: Table S1 — Substrates for nuclease activity and binding assays. (DOCX) [file pbio.1001803.s010.docx]

**Table S1. Substrates for nuclease activity and binding assays.**

| **Nuclease activity assay in Figure 2** | | |
| --- | --- | --- |
| Substrate | | Sequence |
| Stem-loop DNA-3'ACC | | 5'-GGCCCTCTTTAGGGCC**ACC**-3' |
| Bulge DNA | | 5'-**G**GGCCCTCTTTAGGGCC**ACC**-3 |
| Bubble DNA (I-T) | | 5'-**GT**GGCCCTCTTTAGGGCC**IC**-3 |
| Bubble DNA (I-G) | | 5'-**GG**GGCCCTCTTTAGGGCC**IC**-3 |
| Y structured DNA-3'ACC | | 5'-**TTAA**GGCCCTCTTTAGGGCC**ACC**-3' |
|  | | |
| **Nuclease activity assay and gel shift assay in Figure 5 and Supplementary Figure 6** | | |
| Substrate | | Sequence |
| ssDNA 11mer | | 5'-AATCTTACAAA-3' |
| ssDNA 11mer with damaging DNA base (X) | | 5'-GAGTCCTATA **X**-3' |
| Stem-loop DNA | 0 | 5'-GGCCCTCTTTAGGGCC-3' |
|  | 2 | 5'-GGCCCTCTTTAGGGCC**TT**-3' |
|  | 4 | 5'-GGCCCTCTTTAGGGCC**TTAA**-3' |
|  | 6 | 5'-GGCCCTCTTTAGGGCC**TTATAA**-3' |
|  | 10 | 5'-GGCCCTCTTTAGGGCC**AAGGGTTTAA**-3' |
| Y structured DNA | | 5'-**TTAA**GGCCCTCTTTAGGGCC**AAGG**-3' |
