# Supplementary material for: Structural Insights Into DNA Repair by RNase T—An Exonuclease Processing 3′ End of Structured DNA in Repair Pathways
Source: PLoS Biol. 2014 Mar 4;12(3):e1001803. doi: 10.1371/journal.pbio.1001803 (PMC3942315; doi:10.1371/journal.pbio.1001803)
Supplement: Table S2 — Crystallization conditions of RNase T-Structured DNA complexes. (DOCX) [file pbio.1001803.s011.docx]

**Table S2. Crystallization conditions of RNase T-Structured DNA complexes.**

| Structure | DNA | Crystallization conditions | Crystal growing time |
| --- | --- | --- | --- |
| **RNase T-bulge DNA**  **complex 1** | 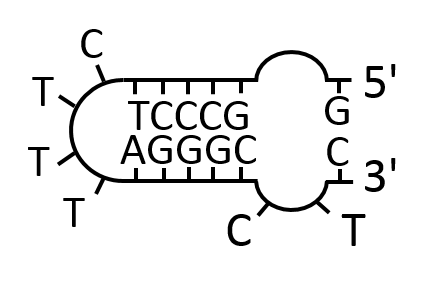 | 0.10% w/v n-Octyl-β-D-glucoside,  0.1 M Sodium citrate tribasic dehydrate pH 5.5,  22% Polyethylene glycol 3350 | 2 weeks - 1.5 month |
| **RNase T-bulge DNA**  **complex 2** | 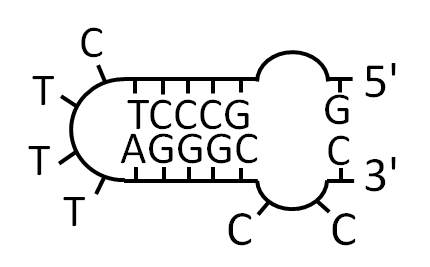 | 6% v/v Tacsimate pH 6.0,  0.1 M MES monohydrate pH 6.0,  25% w/v Polyethylene glycol 4000 | 2 weeks - 1 month |
| **RNase T-Y structured DNA**  **complex** | 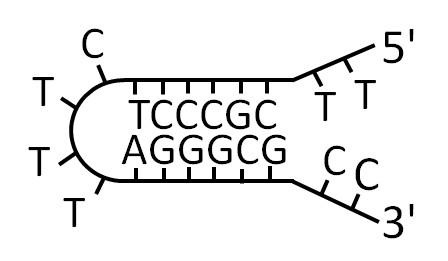 | 20% v/v 2-Propanol,  0.1 M MES monohydrate pH 6.0,  20% w/v Polyethylene glycol monomethyl ester 2000 | 2 weeks - 1 month |
